# Treatment of Young Children with HIV Infection: Using Evidence to Inform Policymakers

**DOI:** 10.1371/journal.pmed.1001273

**Published:** 2012-07-24

**Authors:** Andrew J. Prendergast, Martina Penazzato, Mark Cotton, Philippa Musoke, Veronica Mulenga, Elaine J. Abrams, Diana M. Gibb

**Affiliations:** 1 Centre for Paediatrics, Blizard Institute, Queen Mary University of London, London, United Kingdom; 2 MRC Clinical Trials Unit, London, United Kingdom; 3 Zvitambo Project, Harare, Zimbabwe; 4 Children's Infectious Disease Clinical Research Unit, Faculty of Health Sciences, Stellenbosch University and Tygerberg Children's Hospital, Tygerberg, South Africa; 5 Mulago Hospital, Paediatric Infectious Diseases Clinic, Kampala, Uganda; 6 University Teaching Hospital, Lusaka, Zambia; 7 ICAP, Mailman School of Public Health and College of Physicians & Surgeons, Columbia University, New York, New York, United States of America

## Abstract

Andrew Prendergast and colleagues consider the evidence for a change in policy for the treatment of young children infected with HIV.

**Summary Points**

- Early initiation of antiretroviral therapy (ART) in infants with HIV leads to a 4-fold reduction in mortality compared to deferred ART
- Young children starting a first-line ART regimen containing a non-nucleoside reverse transcriptase inhibitor (nevirapine; NVP) have a 2-fold higher risk of treatment failure than those who start a regimen containing a protease inhibitor (lopinavir/ritonavir; LPV/r)
- Use of LPV/r in infants is challenging due to its expense, unpalatable formulation, and potential long-term toxicity
- Better formulations of ART are urgently required for infants and young children

Despite efforts to scale up prevention of mother-to-child transmission (PMTCT) of HIV, over 1,000 infants continue to be infected daily, particularly in sub-Saharan Africa [1]. Disease progression in infants is much more rapid than in older children and adults, with mortality exceeding 50% by 2 years of age in the absence of antiretroviral therapy (ART) [2]. Although combination ART has been available since 1997, diagnosis and treatment of infants is much more challenging compared to older children and adults (Box 1). Furthermore, until recently there was little evidence to guide treatment approaches in infants and young children, with international policymakers relying on data from cohort studies and expert opinion to inform guidelines. In the past 5 years, results have emerged from several randomized clinical trials of children with HIV under 2 years of age (Table 1) [3]–[8]; a systematic review of these trials has just been published [9]. Here, we consider the implications of research findings for forthcoming World Health Organization (WHO) guidelines and, ultimately, for policymakers, who will need to weigh efficacy and feasibility of interventions in their particular settings in low- and middle-income countries (LMIC).

**Box 1. Challenges in the Treatment of Infants and Young Children with HIV**

- Virological testing is required to ascertain HIV infection status
- Identification of infected infants is frequently delayed
- Disease progression is rapid, with mortality peaking in the first few months of life
- No reliable markers to predict rapid disease progression
- Limited repertoire of antiretroviral drugs and drug formulations
- Liquid formulations expensive, unpalatable, and difficult to carry/store
- Pharmacokinetics variable, due to developing metabolic pathways
- Frequent adjustment in dosing is required due to rapid growth during infancy
- Need for strategic drug sequencing in the context of lifelong treatment
- Adherence is challenging, with reliance on caregivers to administer medication
- Risk of drug resistance due to high viral loads during infancy
- Potential long-term toxicity of treatment, as ART is started during a developmentally sensitive period of early life

**Table 1:** Published randomized clinical trials evaluating treatment strategies in infants and young children with HIV.

Question	Trial Details	Main Results
**When should antiretroviral therapy be started in young children?**	• **CHER** (South Africa) [8]: 377 asymptomatic HIV-infected infants aged 6–12 weeks, with CD4≥25%, were randomized to 1) Immediate ART for 40 weeks; 2) Immediate ART for 96 weeks; 3) Deferred ART according to WHO criteria. First-line ART regimen comprised ZDV, 3TC, LPV/r. Primary outcomes: time to death or failure of first-line ART (defined as failure to reach CD4≥20% by week 24 of ART; decrease to CD4<20% after week 24; progression to CDC severe stage B or stage C clinical events; toxicity requiring >1 drug substitution within the same class, a switch to a new class, or permanent discontinuation of treatment).• **PEHSS** (South Africa) [7]: 63 infants with HIV were enrolled at birth to a pilot feasibility study of early ART strategies, with randomization to: 1) Immediate ART for 1 year; 2) Immediate ART for 12–18 months with up to 3 structured treatment interruptions; 3) Deferred ART according to WHO criteria. First-line ART regimen comprised ZDV, 3TC, NVP, and NFV, with NVP discontinued once virological suppression (VL<50 copies/mL) achieved. Primary outcome: proportion of infants progressing to AIDS by 3 years of age (not yet reported). Clinical, virological, and immunological outcomes have been reported.	• After median follow-up of 40 weeks (IQR 24–58), mortality was 16% in the deferred ART group vs 4% in the early ART groups (*p*<0.001). Early ART was associated with 76% reduction in mortality and 75% reduction in disease progression. The DSMB recommended early dissemination of these findings and urgent evaluation of untreated infants in the deferred group for possible initiation of ART.• No significant difference in 12-month mortality between immediate (12%) and deferred (5%) groups (*p* = 0.65). However, study not designed or powered to address the question of when to start ART. Infants randomized to immediate compared to deferred ART had reduced morbidity (median 7 vs 12 illness episodes; *p* = 0.003), but similar virological and immunological response to ART.
**Which antiretroviral therapy should be started in young children?**	**P1060** (South Africa, Zimbabwe, Zambia, Malawi, Uganda, Tanzania, India) [5],[6]: Two parallel trials of first-line treatment regimens in children below 3 years of age who qualified for ART by WHO criteria. Children in **cohort 1** (*n* = 164) had previously been exposed to sd-NVP; children in **cohort 2** (*n* = 288) had not previously been exposed to sd-NVP. Children were randomized to 1) ZDV, 3TC, NVP or 2) ZDV, 3TC, LPV/r. Primary outcome: Treatment failure by 24 weeks, defined as permanent discontinuation of the treatment regimen for any reason, including death, toxic effects, and virological failure (confirmed viral load <1 log_10_ copies/mL below the study-entry level at 12–24 weeks, or confirmed viral load >400 copies/mL at 24 weeks).	In **cohort 1**, more children in the NVP group than the LPV/r-group reached the primary endpoint of treatment failure at 24 weeks (39.6% vs 21.7%, respectively; *p* = 0.02). Similar results were seen in **cohort 2** (40.1% vs 18.6%, for NVP vs LPV/r, respectively; *p*<0.001). In meta-analysis, the hazard of treatment failure was 2.01 (95% CI 1.47, 2.77) times higher for children starting ART with an NVP-based regimen compared to a LPV/r-based regimen, with no heterogeneity across the two trials.
**Can antiretroviral therapy be switched in young children?**	**NEVEREST** (South Africa) [3],[4]: 323 children under 2 years of age, previously exposed to sd-NVP, started a first-line ART regimen comprising d4T, 3TC, LPV/r. 195 infants who maintained viral load <400 copies/mL for ≥3 months were randomized to 1) continue LPV/r or 2) change to NVP. Primary outcome: Viral load >50 copies/mL at 52 weeks. Safety endpoint: Confirmed viral load >1,000 copies/mL.	Children changing to NVP, compared to those staying on LPV/r, had a lower hazard of VL>50 copies/mL (HR = 0.62, 95% CI 0.41, 0.92; *p* = 0.02), but a higher hazard of confirmed VL>1,000 copies/mL (HR = 10.19, 95% CI 2.36, 43.94; *p* = 0.002). Longer follow-up to 156 weeks confirmed these findings.

ZDV, zidovudine; 3TC, lamivudine; NVP, nevirapine; LPV/r, lopinavir/ritonavir; NFV, nelfinavir; d4T, stavudine; CDC, Centers for Disease Control; DSMB, Data and Safety Monitoring Board.

## When Should Antiretroviral Therapy Be Started in Young Children?

Several small, observational studies suggested a benefit to starting ART early in infants with HIV [10]–[12], but given the challenges of treatment at this age, together with lack of robust evidence, WHO, European, and United States treatment guidelines differed in their recommendations until 2007, when the Children with HIV Early Antiretroviral Therapy (CHER) trial provided definitive evidence of the need to start ART soon after birth [8]. Asymptomatic, immunologically intact infants with HIV recruited before 12 weeks of age were randomized to start ART either immediately, or once clinical or immunological recommended thresholds were reached. Infants starting immediate ART had a 4-fold reduction in mortality and disease progression compared to infants starting deferred ART (Table 1).

Although the CHER trial was conducted in South Africa, it changed policy in all settings, supported by observational data from Europe and the US [10]–[12]. Interim WHO guidance in 2008 recommended early treatment for infants (children under 1 year) in LMIC; in WHO 2010 guidelines, this recommendation was further extended to include all children under 2 years of age. This age extension was not based on new data, but on recognition that, first, the risk of disease progression and mortality remains high between 1 and 2 years of age; second, immunological markers are poorly predictive for clinical progression in young children; and, third, ART initiation improves retention in care [13].

## Adoption of Early Treatment and Barriers to Its Implementation

Many countries were quick to adopt this guidance, but there are considerable barriers to implementation of early treatment. In particular, early ART initiation relies upon early infant diagnosis (EID) of HIV infection. Ideally, a continuum of care should connect PMTCT, EID, and infant ART services, but in reality there are frequently poor linkages within this cascade and high drop-out rates at each step. Current guidelines [13] recommend that EID is undertaken at 4–6 weeks of age in infants born to mothers with HIV. However, most women do not know their HIV status, because of late or incomplete antenatal care and suboptimal access to HIV testing and PMTCT services [1]. Furthermore, HIV diagnosis in infants is more complex than in older children and adults, because it entails virological testing, which remains expensive, labour-intensive, and technically challenging compared to serological testing. Adoption of early treatment guidelines for young children will therefore only have impact if the entire PMTCT/infant care pathway is strengthened. With increased antenatal HIV testing and PMTCT ART coverage, the vast majority of paediatric infections could be prevented; however, even in this situation, those infants who do become infected will largely be born to women with unknown HIV status. For example, with 95% uptake of antenatal testing and 95% PMTCT ART coverage, there will be 2- to 3-fold more infants with HIV born to mothers with unknown HIV status; this rises to 15- to 20-fold if PMTCT ART coverage is only 50% [14].

Although early infant ART is cost-saving compared to deferred ART [15],[16], the relative treatment cost per child is higher than for adults because of the current price of appropriate formulations and the need for lifelong treatment. Unfortunately, where there are competing demands for limited funds and resources, early treatment of infants with HIV is often therefore seen as a high-burden activity. Initiation of the youngest children on ART has lagged unacceptably behind that of older children and adults because of the challenges of treatment at this age (Box 1).

## Which ART Should Be Started in Young Children?

The choice of first-line ART regimen for infants with HIV is frequently complicated by prior exposure to non-nucleoside reverse transcriptase inhibitor (NNRTI) drugs, commonly used in PMTCT programs. A single point mutation in the virus confers high-level resistance to nevirapine (NVP), which is a preferred first-line drug in young children due to its cost, tolerability, inclusion in fixed-dose combination (FDC) tablets, and ease of administration. The only viable alternative for infants and young children in LMIC is to use lopinavir/ritonavir (LPV/r), a protease inhibitor (PI), in place of NVP. The P1060 trial, undertaken in six African countries, compared first-line NVP-based or LPV/r-based ART in young children (<3 years of age) who either had (cohort 1) or had not (cohort 2) been exposed to sd-NVP prophylaxis (Table 1) [5],[6].

Results from cohort 1 were consistent with prior observational data [17], with a higher rate of principally virological treatment failure among children starting a NVP- compared to a LPV/r-based regimen [5]. More surprisingly, children in cohort 2, who had not been exposed to sd-NVP as far as could reasonably be ascertained, and were nearly a year older, showed a similar difference in outcome between first-line regimens [6]. The reasons underlying this difference have been debated, but remain unclear.

## Should Guidelines on ART for Infants and Young Children Be Changed?

WHO guidelines were amended in 2010 following the P1060 cohort 1 results, to recommend LPV/r-based first-line regimens for children under 24 months with previous exposure to NNRTIs for PMTCT [13]. A NVP-based regimen remains the recommended first-line regimen for infants and young children without previous exposure to NNRTIs. What should policymakers do in light of new data from P1060 cohort 2? The findings appear compelling: a 2-fold increased risk of treatment failure if an infant's first-line regimen contains NVP, rather than LPV/r, whether or not the child has previously been exposed to NVP for PMTCT. However, there are advantages and disadvantages to changing guidelines to recommend universal LPV/r-based ART in young children, and the issues discussed below need to be considered.

### Does the Evidence Support a Change in Policy?

The P1060 trial was a robust, timely, and well-conducted study, in a field where few randomized controlled trial data exist; the results were highly significant with no heterogeneity across the two cohorts. However, treatment failure was a composite endpoint; there were very few deaths and the difference between NNRTI and PI arms was mostly driven by virological failure and toxicity. The virological endpoint was also composite (Table 1) and relatively short-term (24 weeks); might longer follow-up have led to a different result? Results from P1060 contrast with longer-term data from other settings. Data from a European/US trial (PENPACT-1), which followed 266 children with HIV (of whom 68 were <3 years) randomized to first-line PI- or NNRTI-based ART for median 5 years [18], and data from an observational cohort of 437 European infants starting either PI- or NNRTI-based ART [19] and followed for median 5.9 years, both showed no significant difference in virological outcome between PI and NNRTI groups. In fact, in the European infant cohort, a four-drug regimen that included NVP and 3 NRTI drugs was associated with better virological suppression and CD4 recovery than a three-drug PI-based regimen [19]. However, findings from Europe and the US cannot necessarily be extrapolated to LMIC. The P1060 trial certainly recruited the most relevant population to address the question of which first-line regimen to use in LMIC and it is unlikely that another trial will address this question. Randomized data from the ARROW trial (http://www.arrowtrial.org; ISRCTN 24791884), which closes this year, will help to inform whether an initial four-drug ART regimen is beneficial in young children.

### What Is the Feasibility of Starting Young Children on PI-Based Regimens?

LPV/r, the only widely available PI, is more challenging to use in infants than is NVP. LPV/r is currently only available as a bitter-tasting liquid, which requires a cold chain for transport, or as a paediatric heat-stable tablet that cannot be crushed as it rapidly loses bioactivity [20]. LPV/r is generally less well tolerated than NVP and children tend to gain weight less well [3],[5]. Recent reports have described cardiac toxicity and transient adrenal dysfunction in newborns [21],[22]; however, the long-term toxicity of LPV/r-based regimens in children starting treatment very early is poorly documented. Although NVP has well described toxicity, characterized by rash, Stevens Johnson syndrome, and/or hepatotoxicity, permanent discontinuation of treatment in infants and young children due to side effects is uncommon [23].

LPV/r has the advantage of a higher genetic barrier to resistance than NVP. In the CHER trial, only 7/375 (2%) infants starting first-line LPV/r-containing ART switched to second-line regimens after median 4.8 years of follow-up [15]. Use of LPV/r may therefore obviate the need for viral load monitoring, especially since it is unclear which second-line regimen could feasibly be used in LMIC following treatment failure with LPV/r, as NNRTI-based regimens would be unlikely to be robust. Darunavir (another PI) retains activity after failure of LPV/r, but requires separate ritonavir boosting and has not been evaluated in young children because of animal toxicity data. Integrase inhibitors or newer NNRTI drugs may be suitable for second-line regimens in children, but are not yet available.

### What Would Be the Cost Implications?

LPV/r-based regimens are approximately 3-fold more expensive than NVP-based regimens (Clinton Healthcare Access Initiative, unpublished data). This is a major consideration in the current context of limited resources and funding uncertainty for ART programs. However, use of a more expensive regimen can be cost-effective and appropriate economic evaluations should be undertaken. The OCTANE trial reported superior outcomes among NVP-exposed women who subsequently initiated a PI-based regimen, compared to an NNRTI-based regimen [24]. Despite a 12-fold increase in cost of an adult LPV/r regimen compared to a NVP regimen, first-line LPV/r was shown to be very cost-effective in women previously exposed to sd-NVP [25].

It appears intuitive that changing guidelines to recommend universal LPV/r-based ART would increase the number of children starting LPV/r. However, in light of the current drive to eliminate new HIV infections in children [26], an increasing number of mothers and infants will access PMTCT interventions, which are primarily NNRTI-based. As PMTCT coverage increases, those infants who do become infected will be eligible to start LPV/r as a result of prior NNRTI exposure, and the additional proportion of infants starting LPV/r due to a change in guidelines will fall as PMTCT coverage increases.

### Treatment 2.0 and the Need for Equity

The WHO-led “Treatment 2.0” strategy was developed to improve the efficiency and impact of HIV care and treatment programs in LMIC [27]. Because health care workers in lower-level facilities manage adults and children together, there is a strong justification for harmonization with adult treatment as much as possible. In this context, universal LPV/r-based first-line regimens may limit the decentralization of paediatric services that is critically needed to narrow the treatment gap between adults and children [1]. The roll-out of LPV/r-based first-line regimens for infants in South Africa, while logistically feasible in most areas, has highlighted the challenges in ensuring cold-chain delivery and establishing effective procurement and supply chains down to primary care settings.

## Better Antiretroviral Drugs Are Needed for Infants and Young Children

Children with HIV in LMIC are a largely neglected population, particularly in terms of access to suitable formulations [28]. There is an urgent need to produce formulations of current drugs that are suitable for young children, particularly FDC tablets that can be dispersed or crushed and mixed with food or liquids (Box 2). The most widely prescribed NNRTI-based regimens are available as FDCs; however, there are currently no PI-based FDCs and, due to the formulation of LPV/r, full FDC regimens are extremely unlikely to be developed. Given the findings of the P1060 trials, development of better PI-based regimens is a high priority. The CHAPAS-2 trial (ISRCTN 01946535) is evaluating the pharmacokinetics and acceptability of a new heat-stable “sprinkle” formulation of LPV/r for young children. If this trial demonstrates acceptability and equivalence of sprinkles to existing formulations, then LPV/r would be much easier to use in young children.

**Box 2. Requirements for Antiretroviral Drugs in Infants and Young Children**

**Appropriate formulation**

- No cold-chain requirements
- Palatable, easy to carry, store, and administer
  1. Fixed-dose combination tablets (scored, crushable, dispersible, chewable)
  2. For PI regimens, heat-stable sprinkles which can be given with food
- Easy to increase doses using weight bands as children grow

**Low toxicity**

- Avoid need for routine laboratory toxicity (biochemistry, haematology) monitoring
- Reduce switching to alternative regimens because of side effects

**Affordable**

**Once-daily dosing**

- To improve adherence and reduce the risk of drug resistance

**Suitable for use with tuberculosis treatment regimens**

- Rifampicin causes drug interactions due to induction of hepatic cytochrome P450.

## Alternative Approaches

An alternative approach to long-term PI treatment, explored in the South African NEVEREST trial (Table 1), is to start all infants on LPV/r, and later substitute NVP [3],[4]. Children changing to NVP in NEVEREST were more likely to maintain an undetectable viral load (<50 copies/mL) than those staying on LPV/r, perhaps because of better adherence to the more palatable NVP formulation. However, children changing to NVP were also more likely to have episodes of higher virological failure (>1,000 copies/mL) if they did fail, compared to those staying on LPV/r, likely because NVP has a low genetic barrier to resistance.

Would this strategy be practical for infants in LMIC? Virological monitoring before and after changing to NVP is not a realistic approach because access to viral load testing remains limited and costly. A simplified approach, using a fixed duration of LPV/r treatment in early infancy then switching to NVP once adherence becomes more challenging beyond infancy, is more practical to implement; however, this strategy still requires better and more affordable PI formulations to be feasible.

The current recommendation for universal, lifelong treatment may be difficult to sustain in LMIC because of cost, long-term toxicity, and eventual likelihood of virological failure. An alternative approach would be to start early ART during infancy, then stop therapy when risk of disease progression is lower. In CHER, around one-third of children who received early ART for 2 years and then stopped had no clinical or immunological indication to restart ART by the end of follow-up at around 5 years of age [15]. However, these children all started ART very early with high CD4 counts, and in a smaller Kenyan study in which infants started ART later, interruption appeared much less feasible [29]. Nonetheless, there is a rationale for further exploring this approach in children, provided treatment interruption can be sufficiently long to deliver worthwhile reductions in toxicity and cost, and there is capacity to closely monitor children off ART.

## Where to Next?

While elimination of childhood infection is the most important goal, it is unclear what will be achievable by 2015 [26]. Treatment guidelines will be revised for children with HIV in 2013; before then, it will be important to critically consider current and forthcoming data. Young children are already the most high-risk and neglected group affected by the HIV epidemic; only 23% of children in need of ART in LMIC were receiving treatment in 2010 [1]. Recommendations must therefore balance evidence with feasibility, and policymakers must continue to advocate a pipeline of appropriate paediatric antiretroviral drugs to enable evidence to be put into practice.

## Abbreviations

ART: antiretroviral therapy
CHER: Children with HIV Early Antiretroviral Therapy
EID: early infant diagnosis
FDC: fixed-dose combination
LMIC: low- and middle-income countries
LPV/r: lopinavir/ritonavir
NNRTI: non-nucleoside reverse transcriptase inhibitor
NVP: nevirapine
PI: protease inhibitor
PMTCT: prevention of mother-to-child transmission
WHO: World Health Organization
